# Acceptability of pre-exposure prophylaxis for HIV prevention: facilitators, barriers and impact on sexual risk behaviors among men who have sex with men in Benin

**DOI:** 10.1186/s12889-020-09363-4

**Published:** 2020-08-20

**Authors:** Carin Ahouada, Souleymane Diabaté, Myrto Mondor, Septime Hessou, Fernand A. Guédou, Luc Béhanzin, Georges Batona, Ndeye Ndiagna Gning, D. Marcel Zannou, Michel Alary

**Affiliations:** 1 Hopital de Zone Allada, Allada, Benin; 2 Centre de recherche du CHU de Québec, Université Laval, Québec, Québec Canada; 3 Département de médecine sociale et préventive, Université Laval, Québec, Québec Canada; 4 Université Alassane Ouattara, Bouaké, Côte d’Ivoire; 5 Centre Interfacultaire de Formation et de Recherche en Environnement pour le Développement Durable (CIFRED/UAC), Abomey-Calavi, Bénin; 6 Dispensaire des IST, Centre de Santé Cotonou I, Cotonou, Bénin; 7 École Nationale de Formation des Techniciens Supérieurs en Santé Publique et en Surveillance Épidémiologique, Université de Parakou, Parakou, Bénin; 8 Centre National Hospitalier et Universitaire Hubert Koutoukou Maga de Cotonou, Cotonou, Benin; 9 Faculté des Sciences de la Santé, Cotonou, Bénin; 10 Institut national de santé publique, Québec, Québec Canada; 11 Hôpital du Saint-Sacrement 1050 chemin Ste-Foy Québec, Québec, QC G1S 4L8 Canada

**Keywords:** Acceptability, PrEP, HIV, MSM, Benin

## Abstract

**Background:**

In Benin, men who have sex with men (MSM) do not always use condoms during anal sex. Pre-exposure prophylaxis (PrEP) using Truvada® (tenofovir disoproxil fumarate / emtricitabine) may be a complementary HIV prevention measure for MSM. This study aimed at identifying the potential facilitators and barriers to the use of PrEP.

**Methods:**

This was a cross-sectional study conducted in 2018 among male-born MSM aged 18 years or older who reported being HIV-negative or unaware of their HIV status. The participants were recruited by the RDS technique (respondent driven sampling) in six cities of Benin. Logistic regression analyses, adapted to RDS statistical requirements, were performed to identify the factors associated with PrEP acceptability.

**Results:**

Mean age of the 400 MSM recruited was 26.2 ± 5.0 years. PrEP was known by 50.7% of respondents. The intention to use PrEP was expressed by 90% of MSM. If PrEP effectiveness were 90% or more, 87.8% of the respondents thought they would decrease condom use. In multivariate analysis, the facilitators associated with PrEP acceptability were: not having to pay for PrEP (odds ratio (OR) = 2.39, 95% CI: 1.50–4.46) and its accessibility within MSM networks (*OR* = 9.82, 95% CI: 3.50–27.52). Only one barrier was significant: the concern that taking PrEP be perceived as marker of adopting HIV risky behaviors (*OR* = 0.11, 95% CI: 0.04–0.30).

**Conclusion:**

In Benin, not all MSM know about PrEP. But once well informed, the majority seems willing to use it if made available. The free availability of the drug and its accessibility in the MSM networks are important facilitators. The possibility of decrease in condom use should not be a barrier to the prescription of PrEP if made available.

## Background

In sub-Saharan Africa, the prevalence of HIV infection among MSM significantly higher among MSM than in the general population [1]. One of the reasons for the high prevalence among MSM may be that anal transmission of HIV without a condom is easier than vaginal transmission without a condom, and individual risks of HIV infection among MSM include unprotected passive anal sex, a high number of male partners, and concurrent injection drug use [2–4].

In Benin, HIV prevalence was estimated at 7.0% among MSM in 2014 [5], compared to 1% [0.7–1.7] in the general population [6]. As observed in other African countries [7], MSM in Benin are known to be difficult to access because they live in hiding due to the extent of stigma and discrimination against them. They have less access to curative and preventive care services. Behavioural prevention measures (regular condom use, abstinence, fidelity and testing) have also shown their limitations. For instance, only 33% of MSM in Benin report having used condoms all the time with all their partners in the last 6 months [5]. In this context of high risk of HIV infection due to the limited means of behavioural prevention, PrEP, integrated to combination prevention programmes, could prove useful in reducing the risk of transmission [8].

PrEP is an oral pill taken daily or on demand to reduce the risk of contracting HIV [9]. According to studies, it reduces HIV acquisition among MSM by 44 to 86% [10–12]. Based on scientific evidence regarding the acceptability of PrEP [13], its cost and feasibility, the World Health Organization (WHO) has expanded its 2014 recommendations to support PrEP supply to all populations at higher risk of HIV [14]. However, PrEP is not yet available for any population in Benin, including MSM. In addition, the scope of PrEP implementation, where available through different programs, appears to depend on its acceptability, the level of knowledge of MSM on this prevention method, as well as on various factors that could influence its use and its impact on risky sexual behaviors. PrEP acceptability varies across studies, with complex reasons in relation to the individual, the provider, the community and the health system [15]. Nearly half of the Nigerian MSM participants in one study had no prior knowledge of PrEP, but after being informed of its potential benefits, 80% were willing to use it [16]. In Kenya, it was found that 64.3% of participants had heard of PrEP and only half were willing to use it [17]. Several barriers were described by authors, such as stigma, cost, frequency of HIV counselling and treatment and possible drug interactions; concerns included the possible abandonment of condoms, increased risk of sexually transmitted infections, and non-compliance with medications and schedules, which need to be taken into account when setting up a PrEP programme [18].

This study aimed to assess PrEP knowledge and acceptability, to identify socio-demographic and behavioral variables associated with these two outcomes, as well as facilitators and barriers, associated with its acceptability in order to gather useful information for the PrEP programme in Benin prior to its implementation.

## Methods

### Participants and procedures

This study focused on men who identify themselves as MSM. Male-born participants, aged 18 years or older, self-reported as being HIV-negative or unaware of their HIV status, who reported at least one anal sex episode with a male partner in the last 12 months, were eligible for participation in this study.

The respondent driven sampling (RDS) technique [19], suitable for this type of population (MSM, hidden population), was used for the recruitment of participants in Benin cities with high concentration of MSM [20] such as Cotonou, Porto Novo, Abomey-Calavi, Pobe, Parakou and Bohicon. The RDS technique uses a referral chain (snowball) methodology for data collection from hard-to-reach populations whose members form linked social networks. RDS starts with a group of participants or “seeds” selected non-randomly from the target population. With the use of appropriate weights, the final sample obtained through RDS can be considered as representative of the target population [19].

In this study, seven seeds were distributed as follows: 2 in Cotonou and 1 in each of the 5 remaining cities. These seven initial seeds were chosen in consultation with the heads of MSM networks and associations in the country so that the seeds would have different characteristics and a large network of friends. After having given written informed consent, the seven seeds participated in the study. Afterward, each of them received five coupons to recruit, each, five MSM who were interested in the study. The new recruits, after having provided their informed consent, were also submitted to the questionnaires and then each received five coupons that allowed them to recruit five new MSM each and so on until the expected number of MSM was reached, our target being of 400 participants.

### Questionnaire

The questionnaire was designed in conjunction with literature data and the results of a preliminary qualitative study on PrEP acceptability among MSM in Benin [21]. The qualitative study served as a springboard to contextualize the quantitative questionnaire used for the present study. This quantitative questionnaire included four sections: size of the personal network; socio-demographic characteristics; HIV-related characteristics and risks; and questions related to PrEP. It was administered through face-to-face interviews by trained investigators using a collection form that was filled in as it was administered. The questionnaire was administered in French and translated into local languages as appropriate. Fixed points were identified in the different cities to which the participants were directed to meet the data collector.

### Measures

Prior to data collection, the questionnaire was pre-tested for understanding with 10 MSM who did not participate in the study afterwards. However, we did not assess test-retest reliability. In addition, since none of the construct used to measure facilitators and barrier to PrEP use included more than one item, the assessment of internal consistency was not applicable. Nearly all the variables described below were extracted from the literature on PrEP acceptability among MSM [22–24], with some variables coming from the qualitative study conducted beforehand [21].

### PrEP knowledge

It was measured by asking participants if they had ever heard of PrEP (yes, no, uncertain). If yes, participants specified the channel through which they had heard about PrEP. Before carrying on with the rest of the questionnaire, we provided the following information about PrEP to all participants: “Pre-exposure prophylaxis (or PrEP) is an HIV prevention method that involves that people without HIV infection take a combination of two antiretroviral drugs in a single pill, also used to treat HIV, on a daily or on demand basis. PrEP is already used in several countries. This HIV prevention method involves uninfected but high-risk people, such as many men who have sex with men. PrEP users take this therapy in anticipation of potential exposure to HIV in order to reduce the risk of infection. Like “birth control pills“ for the prevention of pregnancies, PrEP could therefore be an attractive additional option for at-risk populations”.

### PrEP acceptability

It was defined as the intention to use PrEP. Intention represents the motivation or the will to achieve behavior and is defined as the perception of the probability of adopting a behavior [25]. It has been measured in various ways in the literature, with their own limitations and advantages [23, 26–28]. For this study, a five-point Likert scale ranging from 5 (very likely) to 1 (unlikely) was used. Participants answered the following question: “If PrEP was available in Benin for the prevention of HIV infection, would you intend to use it as a HIV prevention method? ».

### Preference of the desired mode of using PrEP

Participants were successively asked questions about the likelihood for them to use PrEP daily, every 3 days, weekly or on demand. Responses were expressed on a 5-point Likert scale of 5 (very likely) to 1 (not likely). Then they were asked their preference between the daily and the on-demand PrEP use.

### Socio-demographic data

They included: age (years), marital status (married, single, widowed, cohabiting, no answer), education level (out of school, primary, secondary, higher), occupation (pupils or students, salaried employees, artisans or traders, unemployed, others to be specified); religion (traditional, Christian, Muslim, no religion, other), nationality (Beninese, others to be specified), member of an association of MSM (yes, no).

### Sexual characteristics

They included: sexual orientation (homosexual, bisexual, heterosexual), active or insertive sexual role (during the sexual intercourse, who penetrates for anal sex, for oral sex), passive or receptive (during the sexual intercourse, who is penetrated for anal sex, for oral sex), versatile (which changes easily sex role in a sexual relationship, is sometimes insertive, sometimes receptive).

### HIV risk perception

On a 5-point Likert scale ranging from 5 (very high) to 1 (not high at all), participants answered the following question: “Referring to your past and present sexual practices, at which level do you rank your risk of contracting HIV?”

### Sexual behaviors during the last 6 months

These variables included: number of male sexual partners, sex outside the regular relationship, number of unprotected insertive and receptive sexual acts, condom use at last sex with a man, frequency of anal intercourse during the last 6 months; sexual relations after consumption of drugs or alcohol, sex in exchange for money or gifts, number of female partners, condom use during last sexual intercourse with a woman.

### Facilitators and barriers to PrEP use

Facilitators and obstacles were identified in the literature [24], and then supplemented by other factors identified in the qualitative study carried out prior to this quantitative study [21].

The following question was asked: How important do you consider the following statements to be as facilitators or barriers to the use of PrEP? (1 = not at all important, 2 = not important, 3 = neutral, 4 = important, 5 = very important).

The facilitators used in this study were: 1. not having to pay for PrEP, 2. access to free HIV testing, 3. access to free health care/sexual monitoring, 4. access to individual support and support around the use of PrEP, 5. access to information on the use of PrEP, 6. access to support or counseling on my sex life, 7. not having to go to the casual doctor for the PrEP, 8. access to group membership information on PrEP use, 9. drug availability, 10. drug accessibility at the level of MSM network, 11. self-protection concern, 12. possibility of multiple partnerships, 13. lack of constraints during drug procurement, 14. sex possibilities with HIV-Positive.

The barriers used were: 1. concerns about PrEP long-term effects on my health, 2. concern about the fact that if I become infected by HIV, some ARV will no longer be efficient because they would have been taken as PrEP, 3. concern about the fact that PrEP does not provide a complete protection against HIV, 4. taking a drug every day, 5. concern that taking PrEP could make me more likely to have sex without condom, 6. concerns that having to take PrEP means that I put myself at risk for HIV, 7. PrEP could make my partners expect to have anal sex without condom with me, 8. concerns that people will see me taking medication and think I have HIV, 9. concerns that people will see me taking drug and will want to know why I’m taking it, 10. having to talk to my doctor about my sex life, 11. binding procedures for the drug procurement, 12. size and taste of the medication, 13. fee-paying drug, 14. concern that PrEP may lead to prostitution, 15. concern that PrEP might encourage to be unfaithful, 16. partner’s disagreement because I’m taking PrEP, 17. unreceptive attitude of the MSM community towards PrEP, 18. PrEP as source of discrimination in health centers, 19. concern that PrEP may increase risk-taking (e.g., increase in the number of unprotected sex acts, increase in the number of sexual partners, etc.), 20. concern that PrEP may increase the risk of contracting sexually transmitted infections other than HIV.

### Risk behaviors under PrEP

On a five-point Likert scale ranging from 5 (definitely increase) to 1 (definitely decrease), participants answered the following three questions: according to you, how could PrEP use with a 90% effectiveness affect your frequency of condom use during anal sex? According to you, how could the use of PrEP with 90% effectiveness affect the number of your male sexual partners? According to you, how could the use of PrEP with 90% effectiveness affect the number of your anal intercourses?

### Data processing

Data were entered into an EPI Data Version 3.1 database [29]. The variables measured on the Likert scale were dichotomized depending on their distribution across the five categories. Conceptually, categorization (4 + 5) versus (1 + 2 + 3) for the dependent variable (acceptability) is preferred. However, it was decided to use (5) versus (1 + 2 + 3 + 4) because there were not enough participants with (1 + 2 + 3). Different categorizations of the independent variables were tried. The categorization (4 + 5) versus (1 + 2 + 3) was chosen when possible (8 variables are categorized as (5) versus (1 + 2 + 3 + 4)). This seemed preferable from a conceptual point of view, as it provided reasonable « n » for the categories and logical results. Variables with low effective frequencies in categories 3, 2, and 1 were dichotomized as 5 versus 4 + 3 + 2 + 1. This includes the dependent variable (acceptability) and the following potential facilitators: not having to pay for PrEP, access to free HIV testing, access to free health care / sexual surveillance, access to personal coaching and support around the use of PrEP, access to information on the use of PrEP, access to support or counseling about one’s sex life, availability of the drug, accessibility of the drug at MSM level. The other variables measured on a Likert scale were dichotomized as 5 + 4 versus 3 + 2 + 1.

### Statistical analysis

Data analysis used the RDS Analyst statistical software, version 0.65 [30] and SAS, version 9.4 (SAS Institute Inc., Cary, NC, USA). The RDS Analyst software was used to generate probability weights for each observation that were used in both the univariate and multivariate analyses with SAS. RDS-II weights calculated from the participant’s network size were used in order to address the potential biases introduced by chain recruitment [31]. Some extreme values were reported by the participants ​​ for the network sizes. To overcome this problem, we used an approach proposed by the RDS Analyst statistical software [32], which consists in calculating the weights with truncated network sizes. All network sizes above the 95th percentile were reduced to the 95th percentile (all sizes ≥110 were reduced to 110). And all network sizes below the 5th percentile were adjusted to the 5th percentile value (all sizes ≤4 were adjusted to 4).

Continuous variables were expressed in means with their standard deviation or medians with their interquartile ranges. Logistic regression was used to identify factors associated with knowledge and PrEP acceptability [33]. For knowledge of PrEP, the multivariate model was developed with the socio-demographic and behavioral variables selected from the univariate analysis at a threshold of *p* < 0.20. For PrEP acceptability, a first multivariate model was developed with the facilitators and barriers variables selected from the univariate analysis at a threshold of *p* < 0.20. A descending manual procedure was used to obtain an intermediate model at a threshold of *p* < 0.05. The socio-demographic and behavioral variables retained in the univariate analysis at a threshold of *p* < 0.20 were then added to this intermediate model. A new descending manual procedure was then performed in order to achieve the final model at a threshold of *p* < 0.05. The SAS survey logistic procedure was used for these univariate and multivariate analyses. The truncated weights generated by RDS Analyst served as the probability weights [34] and seeds were used as clusters to take into account homophily (the tendency to recruit later participants with the same characteristics as the initial ones). The adjusted odds ratios (OR) and their 95% confidence intervals (95%CI) were calculated.

## Results

**Fig. 1:**
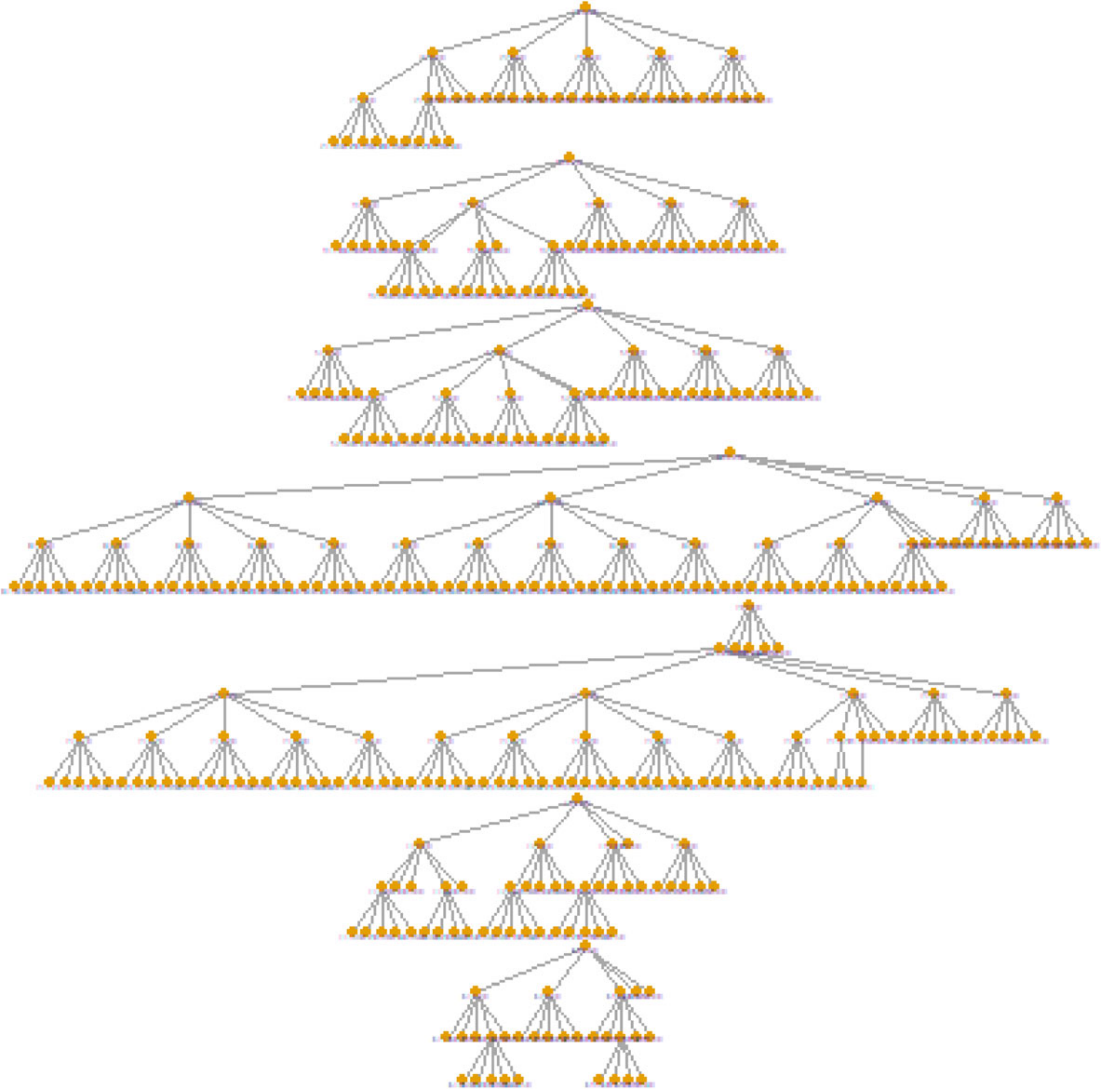
Graphical representation of the recruitment generated by RDS Analyst (7 seeds), Benin, 2017–2018

A total of 400 MSM were interviewed in the six cities covered by the study. Figure 1 presents the recruitment chain for each of the seven seeds.

The population consisted of young people [mean age (standard deviation) 26.2 (5.0) years], mostly single (85.5%) with at a least secondary educational level (90.5%) and of Christian religion (57.7%). Those with a homosexual orientation accounted for 64% of the population, the rest of the participants reporting being bisexual. Sex roles in homosexual relationships were active (39.7%), passive (39.7%) or versatile (20.5%). Participants had sex with an average number of four different men in the last 6 months preceding the survey. Condom use at last sex was reported by 76.8% of the participants. Some of the surveyed MSM reported sex in exchange for money or gifts (24.5%).

PrEP was known to 50.7% of respondents before this study. Table 1 shows the univariate and multivariate analyses of the associations between participants’ characteristics and PrEP knowledge. In multivariate analysis, knowledge of PrEP was positively associated with advanced level of education (*OR* = 9.17, 95%CI: 2.41–34.88) and being married compared to being single (*OR* = 3.91, 95%CI: 1.12–13.61). Factors negatively associated with PrEP knowledge were: married status (*OR* = 0.25, 95%CI: 0.07–0.88); common-law union (*OR* = 0.12, 95%CI: 0.02–0.66); sexual intercourse after drug or alcohol use in the last 6 months (*OR* = 0.36, 95%CI: 0.22–0.60); and condom use at last sexual intercourse with a woman (*OR* = 0.44, 95%CI: 0.28–0.68).

**Table 1:** Characteristics of the MSM population and their associations with knowledge of PrEP, Benin

Characteristics	Knowledge about PrEP		OR (95% CI)^**a**^	***p***-value	AOR (95%CI)^**b**^	***p***-value
	Yes ***N*** = 203	No ***N*** = 197				
	n (%)	n (%)				
**Socio-demographic characteristics**						
**Age as a continuous variable, mean (standard deviation)**	26.04 (5)	26.36 (5)	1.06 (0.96–1.17)	0.24		
**Age categories**						
< 20	11 (5.4)	10 (5.1)	1		1	
20–24	75 (36.9)	66 (33.5)	1.01 (0.42–2.42)	0.96	1.53 (0.88–2.65)	0.12
25–29	72 (35.5)	68 (34.5)	1.47 (0.42–5.52)	0.53	1.99 (0.83–4.77)	0.11
30–34	28 (13.8)	39 (19.8)	1.36 (0.19–9.50)	0.75	1.95 (0.35–10.37)	0.45
≥ 35	17 (8.4)	14 (7.1)	3.74 (0.62–22.63)	0.14	3.53 (0.89–13.99)	0.07^¶^
**Marital Status**						
Single	170 (83.8)	172 (87.3)	1		1	
Divorced or widowed	4 (2.0)	5 (2.5)	2.29 (0.50–10.33)	0.27	0.63 (0.19–2.02)	0.43
De facto union	7 (3.4)	13 (6.6)	0.41 (0.10–1.64)	0.20	0.47 (0.18–1.17)	0.10
Married	22 (10.8)	7 (3.6)	**5.50 (1.87–16.19)**	**0.002**	**3.91 (1.12–13.61)**	**0.03**
**Level of Education**						
Primary or less	9 (4.4)	29 (14.7)	1		1	
Secondary	81 (39.9)	132 (67.0)	**3.06 (1.04–8.94)**	**0.04**	2.99 (0.81–10.97)	0.09
Advanced	113 (55.7)	36 (18.3)	**11.32 (3.86–33.18)**	**<.0001**	**9.17 (2.41–34.88)**	**0.001**
**Work situation**						
Pupils/Students	103 (50.7)	52 (26.4)	1			
Salaried employees	46 (22.7)	34 (17.3)	1.04 (0.32–3.31)	0.94		
Craftsmen/Salesmen	26 (12.8)	78 (39.6)	**0.46 (0.26–0.81)**	**0.007**		
Unemployed	12 (5.9)	15 (7.6)	1.48 (0.14–15.39)	0.73		
Others	16 (7.9)	18 (9.1)	0.59 (0.13–2.62)	0.48		
**Religion**						
Traditional	15 (7.4)	17 (8.5)	1			
Christianity	123 (60.5)	108 (54.8)	0.93 (0.22–3.89)	0.92		
Islamism	59 (29.1)	59 (29.9)	1.06 (0.20–5.50)	0.94		
No religion	5 (2.5)	12 (6.1)	1.07 (0.34–3.33)	0.89		
Others	1 (0.5)	1 (0.5)	10.84 (0.87–134)	0.06		
**Site of data collection**						
Cotonou	90 (44.3)	71 (36.0)	1.30 (0.32–5.26)	0.71		
Outside Cotonou	113 (55.7)	126 (64.0)	1			
**HIV-related characteristics and risks**						
**Sexual orientation**						
Homosexual	128 (63.1)	128 (65.0)	1			
Bisexual	75 (36.9)	69 (35.0)	0.69 (0.27–1.720	0.42		
**Sexual Roles**						
Active or insertive	74 (36.4)	85 (43.1)	1			
Passive or receptive	86 (42.4)	73 (37.1)	1.54 (0.84–2.82)	0.15		
Both	43 (21.2)	39 (19.8)	0.90 (0.45–1.82)	0.78		
**Personal HIV-related risk assessment**						
Low (3 + 2 + 1)	189 (93.1)	184 (93.4)	1			
High (5 + 4)	14 (6.9)	13 (6.6)	0.92 (0.13–6.21)	0.93		
**Average number of men with whom sex has occurred during the last 6 months**	4.33 (3.38)	3.95 (2.93)	1.08 (0.93–1.25)	0.26		
**Sex with regular male partners during the last 6 months**						
No	22 (10.8)	45 (22.8)	1			
Yes	181 (89.2)	152 (77.2)	1.56 (0.73–3.29)	0.24		
**Average number of insertive intercourses during the last 6 months**	7.93 (11.23)	11.4 (13.36)	0.96 (0.90–1.01)	0.17		
**Average number of receptive intercourses during the last 6 months**	9.87 (10.49)	11.46 (11.91)	0.99 (0.94–1.05)	0.97		
**Number of unprotected insertive intercourses during the last 6 months**	1.78 (3.68)	2.94 (4.58)	**0.88 (0.78–0.99)**	**0.04**		
**Number of unprotected receptive intercourses during the last 6 months**	2.19 (3.89)	3.29 (5.33)	0.93 (0.85–1.01)	0.10		
**Condom use during the last sex activity with a man**						
No	35 (17.2)	58 (29.4)	1			
Yes	168 (82.8)	139 (70.6)	**4.30 (1.71–10.79)**	**0.001**		
**Number of anal intercourses during the last 6 months**	10.89 (10.47)	13.73 (12.99)	0.97 (0.92–1.01)	0.24		
**Sex after consumption of drug or alcohol during the last 6 months**						
No	115 (56.6)	85 (43.2)	1		1	
Yes	88 (43.4)	112 (56.8)	**0.44 (0.27–0.74)**	**0.002**	**0.36 (0.22–0.60)**	**< 0.0001**
**Sex in exchange for money or gifts during the last 6 months**						
No	161 (79.3)	141 (71.6)	1			
Yes	42 (20.7)	56 (28.4)	0.62 (0.11–3.49)	0.59		
**Average number of women with whom sex occurred during the last 6 months**	0.79 (1.52)	0.69 (1.71)	0.97 (0.84–1.13)	0.77		
**Condom use during the last sexual intercourse with a woman (*n*** **= 225)**						
No	55 (51.9)	77 (64.7)	1		1	
Yes	51 (48.1)	42 (35.3)	1.62 (0.95–2.77)	0.07	**0.44 (0.28–0.68)**	**0.0003**

^a^Weighted odds ratio (probability weights generated by RDS Analyst)

^b^Weighted adjusted odds ratio (probability weights generated by RDS Analyst)

^¶^*p* = 0.30, test for linear trend of the association between age and PrEP knowledge; this variable was kept in the model because it was confounding of the other associations

The participants mostly preferred using it on a daily basis (69.2%). If PrEP effectiveness were 90%, most respondents thought they would decrease condom use (87.8%), increase the number of male sexual partners (69.0%), and increase the number of anal sex acts (74.2%). Nine out of ten MSM expressed a high intention (Likert 4 + 5) to use PrEP, including (35.8%) with a very high intention (Likert 5). The average (± standard deviation) of the Likert scale on the intention to use PrEP was 4.2 (± 0.8).

In univariate analysis, as shown in Table 2, acceptability was associated with age group 20–24 compared to < 20 (*OR* = 3.3, 95%CI: 1.12–9.80), with divorced and / or widowed status compared to married (*OR* = 17.4, 95%CI: 2.01–149.91), and with bisexual compared to homosexual orientation (*OR* = 2.8, 95%CI: 1.74–4.64).

**Table 2:** Characteristics of the MSM population and their association with PrEP acceptability, Benin

Characteristics		PrEP Acceptability				
	***N*** = 400	Yes (5) ***N*** = 143	No (4 + 3 + 2 + 1) ***N*** = 257	OR^**a**^	95%CI	***p***-value
		n (%)	n (%)			
**Socio-demographic characteristics**						
**Age as a continuous variable (mean (standard deviation)**	26.20 (5.0)	26. 06(4.8)	26. 28(5.1)	1.01	0.911–1.13	0.75
**Age categories**						
< 20	21(5.3)	5(3.5)	16(6.2)	1		
20–24	141(35.3)	55(38.5)	86(33.5)	3.32	1.12–9.80	**0.02**
25–29	140(35)	51(35.7)	89(34.7)	2.73	0.65–11.42	0.16
30–34	67(16.7)	24(16.7)	43(16.7)	3.93	0.57–26.87	0.16
≥ 35	31(7.7)	8(5.6)	23(8.9)	1.40	0.14–13.57	0.76
**Marital Status**						
Married	29(7.2)	12(8.4)	17(6.6)	1		
Single	342(85.5)	114(79.7)	228(88.7)	1.66	0.45–6.15	0.44
Divorced or widowed	9(2.3)	7(4.90)	2(0.8)	17.38	2.01–149.91	**0.009**
De facto union	20(5)	10(6.9)	10(3.9)	1.83	0.29–11.29	0.51
**Level of Education**						
None	4(1.0)	1(0.7)	3(1.2)	1		
Primary	34(8.6)	13(9.1)	21(8.2)	1.22	0.28–5.29	0.78
Secondary	213(53.2)	88(61.5)	125(48.6)	1.70	0.40–7.11	0.46
Advanced	149(37.2)	41(28.7)	108(42.0)	0.75	0.16–3.47	0.72
**Work situation**						
Pupils/Students	155(38.7)	56(39.2)	99(38.5)	1		
Salaried employees	80(20.0)	24(16.7)	56(21.8)	0.59	0.25–1.39	0.23
Craftsmen/Salesmen	104(26)	26(18.2)	78(30.4)	0.83	0.32–2.15	0.70
Unemployed	27(6.7)	12(8.4)	15(5.8)	1.59	0.21–11.69	0.64
Others	34(8.6)	25(17.5)	9(3.5)	2.97	0.76–11.58	0.11
**Religion**						
Traditional	32(8.0)	15(10.5)	17(6.6)	1		
Christianity	231(57.8)	91(63.6)	140(54.5)	0.65	0.31–1.35	0.25
Islamism	118(29.5)	32(22.4)	86(33.5)	0.48	0.10–2.30	0.36
No religion	17(4.2)	4(2.8)	13(5.1)	0.55	0.12–2.52	0.44
Others	2(0.5)	1(0.7)	1(0.3)	5.97	0.33–105.48	0.22
**Site of data collection**						
Cotonou	239 (59.7)	88(61.5)	151(58.75)	1.45	0.25–8.26	0.67
Outside Cotonou	161 (40.3)	55(38.5)	106(41.25)	1		
**Knowledge about PrEP**						
Yes	203(50.75)	80(55.9)	123(47.9)	0.66	0.16–2.73	0.57
No	197(49.25)	63(44.1)	134(52.1)	1		
**Preferred PrEP usage mode**						
On-demand	123(30.7)	51(35.7)	72(28.0)	1.48	0.83–2.63	0.17
Daily	277(69.3)	92(64.3)	185(71.9)	1		
**HIV-related characteristics and risks**						
**Sexual orientation**						
Homosexual	256(64)	60(41.9)	196(76.3)	1		
Bisexual	144(36)	83(58.1)	61(23.7)	2.85	1.74–4.64	**<.0001**
**Sexual Roles**						
Active or insertive	159(39.8)	56(39.2)	103(40.1)	1		
Passive or receptive	159(39.7)	48(33.6)	111(43.2)	0.64	0.37–1.13	0.12
Both	82(20.5)	39(27.2)	43(16.7)	1.80	0.76–4.27	0.17
**HIV testing during the last 12 months**						
No	8(2.0)	6(4.2)	2(0.8)	1		
Yes	392(98.0)	137(95.8)	255(99.2)	0.42	0.13–1.34	0.14
**Personal HIV-related risk assessment**						
Low (3 + 2 + 1)	373(93.3)	125(87.4)	248(96.5)	1		
High (5 + 4)	27(6.7)	18(12.6)	9(3.5)	2.54	0.61–10.51	0.19
**Average number of men with whom sex has occurred during the last 6 months**	4.15 (3.1)	3.91 (3.9)	4.26 (2.6)	0.93	0.85–1.03	0.17
**Sex with regular male partners during the last 6 months**						
No	67(16.7)	19(13.3)	48(18.7)	1		
Yes	333(83.2)	124(86.7)	209(81.3)	1.19	0.36–3.85	0.76
**Average number of insertive intercourses during the last 6 months**	9.59 (12.4)	8.95 (11.6)	9.96 (12.8)	1.01	0.95–1.07	0.70
**Average number of receptive intercourses during the last 6 months**	10.59 (11.2)	8.12 (9.6)	12.19 (11.8)	0.99	0.93–1.05	0.92
**Number of unprotected insertive intercourses during the last 6 months**	2.33 (4.2)	2.79 (5.1)	2.06 (3.4)	1.06	0.95–1.18	0.27
**Number of unprotected receptive intercourses during the last 6 months**	2.69 (4.6)	2.84 (5.5)	2.59 (3.9)	1.05	0.98–1.12	0.12
**Condom use during the last sex activity with a man**						
No	93(23.3)	48(33.6)	45(17.5)	1		
Yes	307(76.7)	95(66.4)	212(82.5)	0.46	0.14–1.46	0.18
**Number of anal intercourses during the last 6 months**	12.28 (11.8)	14.37 (11.1)	11.12 (12.0)	1.07	0.95–1.21	0.23
**Sex after consumption of drug or alcohol during the last 6 months**						
No	200(50.0)	74(51.7)	126(49.1)	1		
Yes	200(50.0)	69(48.3)	131(50.9)	1.22	0.45–3.30	0.68
**Sex in exchange for money or gifts during the last 6 months**						
No	302(75.5)	105(73.4)	197(76.6)	1		
Yes	98(24.5)	38(26.6)	60(23.4)	1.60	0.55–4.62	0.38
**Average number of women with whom sex occurred during the last 6 months**	0.74 (1.6)	1.02 (1.4)	0.58(1.7)	1.23	0.92–1.65	0.15
**Condom use during the last sexual intercourse with a woman (*n*** **= 225)**						
No	132(58.7)	57(57.6)	75(59.5)	1		
Yes	93(41.3)	42(42.4)	51(40.5)	1.12	0.53–2.34	0.76
**Sexual behaviors under PrEP**						
**Condom frequency use under PrEP with an effectiveness of 90%**						
Decreased (1 + 2 + 3)	351(87.7)	142(99.3)	209(81.3)	93.57	7.15–999	**0.0006**
Increased (4 + 5)	49(12.3)	1	48(18,7)	1		
**Frequency use of condom under PrEP with a 70% effectiveness**						
Decreased (1 + 2 + 3)	84(21.0)	126(88.1)	190(73.9)	5,04	1.20–21.05	**0.02**
Increased (4 + 5)	316(79.0)	17(11.9)	67(26.1)	1	0.04–0.82	
**Number of sexual male partners under PrEP with a 90% effectiveness**						
Decreased (1 + 2 + 3)	124(31.0)	40(27.9)	84(32.7)	1		
Increased (4 + 5)	276(69.0)	103(72.1)	173(67.3)	0.95	0.45–2	0.89
**Number of anal intercourses under PrEP with a 90% effectiveness**						
Decreased (1 + 2 + 3)	103(25.7)	26(18.2)	77(29.9)	1		
Increased (4 + 5)	297(74.3)	117(81.8)	180(70.1)	1.32	0.49–3.58	0.57

^a^weighted odds ratios (probability weights generated by RDS Analyst)

PrEP’s acceptability facilitators identified during the univariate analysis are shown in Table 3. Most facilitators were perceived as important with almost all averages > 4. However, few barriers were perceived as important with average > 4. The facilitators significantly associated (OR > 1) with a high PrEP acceptability included: not having to pay for PrEP (*OR* = 9.45, 95%CI: 3.36–26.62), access to free HIV test (*OR* = 7.19, 95%CI: 2.90–17.81), Access to free healthcare/sexual life supervision (*OR* = 5.13, 95%CI: 2.24–11.73); Access to individual support and support around the use of PrEP (*OR* = 7.04, 95%CI: 2.65–18.67); Access to information on the use of PrEP (*OR* = 4.65, 95%CI: 1.32–16.37); Access to support or counseling about my sexual life (*OR* = 4.41, 95%CI: 1.80–10.79); PrEP drug availability (*OR* = 9.97, 95%CI: 1.73–57.32), PrEP drug accessibility at the level of MSM networks (*OR* = 15.14, 95%CI: 3.89–58.95), sex opportunity with HIV-positive people (*OR* = 2.09, 95%CI: 1.05–4.16). Barriers identified as significantly associated with acceptability of PrEP (OR < 1) were: the concern that “using PrEP means that I put myself at risk for HIV” (*OR* = 0.16, 95%CI: 0.06–0.41); the concern that “people will see me taking medication and think that I have HIV” (*OR* = 0.33, 95%CI: 0.14–0.77), restrictive procedures for drug procurement (*OR* = 0.34, 95%CI: 0.12–0.93); as well as pill’s size and taste (*OR* = 0.32, 95%CI: 0.11–0.91).

**Table 3:** Univariate analysis of PrEP acceptability according to facilitators and obstacles, Benin

	Average ± standard deviation	Acceptability				
		Yes (5) ***N*** = 143	No (4 + 3 + 2 + 1) ***N*** = 257	OR^**a**^	CI 95%	p
		n(%)	n(%)			
**Facilitators**						
Not having to pay for PrEP	4.48 ± 0.58					
5		116 (81.1)	88(34.3)	9.45	3.36–26.62	**<.0001**
(4 + 3 + 2 + 1)		27 (18.9)	169(65.7)	1		
Access to free HIV testing	4.46 ± 0.49					
5		104(72.7)	82(31.9)	7.19	2.90–17.81	**<.0001**
(4 + 3 + 2 + 1)		39(27.3)	175(68.1)	1		
Access to free healthcare/sexual life supervision	4.45 ± 0.51					
5		98(68.5)	84(32.7)	5.13	2.24–11.73	**0.001**
(4 + 3 + 2 + 1)		45(31.5)	173(67.3)	1		
Access to individual support and support around the use of PrEP	4.40 ± 0.57					
5		100(69.9)	74(28.8)	7.04	2.65–18.67	**<.0001**
(4 + 3 + 2 + 1)		43(30.1)	183(71.2)	1		
Access to information on the use of PrEP	4.61 ± 0.53					
5		115(80.4)	138(53.7)	4.65	1.32–16.37	**0.01**
(4 + 3 + 2 + 1)		28(19.6)	119(46.3)	1		
Access to support or counseling about my sexual life	4.38 ± 0.63					
5		99(69.2)	74(28.8)	4.41	1.80–10.79	**0.001**
(4 + 3 + 2 + 1)		44(30.8)	183(71.2)	1		
Not having to go to the casual doctor for the PrEP	3.78 ± 0.98					
(5 + 4)		113(79.1)	192(74.7)	1.02	0.37–2.82	0.95
(3 + 2 + 1)		30(20.9)	65(25.3)	1		
Access to group memberships information on PrEP use	4.18 ± 0.72					
(5 + 4)		130(90.9)	241(93.8)	0.53	0.21–1.31	0.17
(3 + 2 + 1)		13(9.1)	16(6.2)	1		
Drug availability	4.80 ± 0.40					
5		134(93.7)	190(73.9)	9.97	1.73–57.32	**0.01**
(4 + 3 + 2 + 1)		9(6.3)	67(26.1)	1		
Drug accessibility at the level of MSM networks	4.18 ± 1.01					
5		125(87.4)	73(28.4)	15.14	3.89–58.95	**<.0001**
(4 + 3 + 2 + 1)		18(12.6)	184(71.6)	1		
Self-protection concern	4.18 ± 0.86					
(5 + 4)		126(88.1)	207(80.5)	1.16	0.25–5.32	0.84
(3 + 2 + 1)		17(11.9)	50(19.5)	1		
Possibilities of Multiple partnerships	3.26 ± 1.12					
(5 + 4)		93(65.0)	80(31.1)	1.78	0.56–5.63	0.32
(3 + 2 + 1)		50(35.0)	177(68.89)	1		
Lack of constraints during drug procurement	4.35 ± 0.66					
(5 + 4)		130(90.9)	245(95.3)	0.89	0.52–1.55	0.69
(3 + 2 + 1)		13(9.1)	12(4.7)	1		
Sex possibilities with HIV-Positive	301 ± 1.03					
(5 + 4)		66(46.2)	59(23)	2.09	1.05–4.16	**0.03**
(3 + 2 + 1)		77(53.8)	198(77)	1		
**Obstacles**						
Concerns about PrEP long-term effects on my health	3.87 ± 1.01					
(5 + 4)		99(69.2)	225(87.5)	0.38	0.11–1.29	0.12
(3 + 2 + 1)		44(30.7)	32(12.5)	1		
Concern about the fact that if I become infected by HIV, some ARV will no longer be efficient because they would have been taken as PrEP	3.57 ± 1.06					
(5 + 4)		78(54.5)	151(58.7)	0.47	0.11–1.91	0.29
(3 + 2 + 1)		65(45.5)	106(41.3)	1		
Concern about the fact that PrEP does not provide a complete protection against HIV	3.78 ± 0.99					
(5 + 4)		90(62.9)	205(79.8)	0.51	0.14–1.89	0.31
(3 + 2 + 1)		53(37.1)	52(20.2)	1		
Taking a drug every day	3.53 ± 1.01					
(5 + 4)		83(58.0)	192(74.7)	0.31	0.09–1.03	0.05
(3 + 2 + 1)		60(42.0)	65(25.3)	1		
Concern that taking PrEP could make me more likely to have sex without condom	3.33 ± 0.96					
(5 + 4)		78(54.5)	140(54.5)	0.77	0.17–3.45	0.73
(3 + 2 + 1)		65(45.5)	117(45.5)	1		
Concerns that having to take PrEP means that I put myself at risk for HIV	2.97 ± 1.06					
(5 + 4)		26(18.2)	130(50.6)	0.16	0.06–0.41	**0.002**
(3 + 2 + 1)		117(81.8)	127(49.4)	1		
PrEP could make my partners expect to have anal sex without condom with me	3.47 ± 0.93					
(5 + 4)		84(58.7)	162(64)	0.46	0.11–1.91	0.28
(3 + 2 + 1)		59(41.3)	95(36)	1		
Concerns that people will see me taking drug and will think I have HIV	3.79 ± 1.01					
(5 + 4)		88(61.5)	220(85.6)	0.33	0.14–0.77	**0.01**
(3 + 2 + 1)		55(38.5)	37(14.4)	1		
Concerns that people will see me taking drug and will want to know why I’m taking it	3.67 ± 1.05					
(5 + 4)		74(51.7)	221(86.0)	0.29	0.08–1.05	0.05
(3 + 2 + 1)		69(48.3)	36(14.0)	1		
Having to talk to my doctor about my sex life.	2.89 ± 1.14					
(5 + 4)		60(42.0)	74(28.8)	1.44	0.58–3.57	0.42
(3 + 2 + 1)		83(58.0)	183(71.2)	1		
Binding procedures for the drug procurement	4.19 ± 0.79					
(5 + 4)		117(81.8)	245(95.3)	0.34	0.12–0.93	**0.03**
(3 + 2 + 1)		26(18.2)	12(4.7)	1		
Size and Taste of drug	3.81 ± 1.06					
(5 + 4)		93(65.3)	219(85.21)	0.32	0.11–0.91	**0.03**
(3 + 2 + 1)		50(34.7)	38(14.79)	1		
Fee-paying drug	4.27 ± 0.76					
(5 + 4)		125(87.4)	248(96.5)	0.44	0.15–1.33	0.14
(3 + 2 + 1)		18(12.6)	9(3.5)	1		
Concern that PrEP may lead to prostitution	3.5 ± 1.08					
(5 + 4)		98(68.5)	150(58.7)	1.01	0.18–5.55	0.98
(3 + 2 + 1)		45(31.5)	107(41.3)	1		
Concern that PrEP might encourage to be unfaithful,	3.49 ± 1.13					
(5 + 4)		101(70.6)	149(58.0)	1.29	0.24–6.78	0.75
(3 + 2 + 1)		42(29.4)	108(42.0)	1		
Partner’s disagreement because I’m taking PrEP	3.08 ± 1.11					
(5 + 4)		54(37.8)	138(53.7)	0.28	0.06–1.27	0.10
(3 + 2 + 1)		89(62.2)	119(46.3)	1		
The unresponsive attitude of MSM community towards PrEP,	3.39 ± 1.18					
(5 + 4)		70(49.0)	159(61.9)	0.37	0.07–1.83	0.22
(3 + 2 + 1)		73(51.0)	98(38.1)	1		
PrEP as source of discrimination in health centers	3.75 ± 0.99					
(5 + 4)		93(65.0)	214(83.3)	0.41	0.10–1.74	0.23
(3 + 2 + 1)		50(34.0)	43(16.7)	1		
Concern that PrEP may increase risk-taking (e.g: increase in unprotected sex, number of sexual partners, etc)	3.86 ± 0.96					
(5 + 4)		89(62.2)	227(88.3)	0.36	0.12–1.12	0.07
(3 + 2 + 1)		54(37.8)	30(11.7)	1		
Concern that PrEP may increase the risk of contracting sexually transmitted infections other than HIV	4 ± 0.99					
(5 + 4)		101(70.6)	230(89.5)	0.55	0.14–2.09	0.38
(3 + 2 + 1)		42(29.4)	27(10.5)	1		

^a^weighted odds ratios (probability weights generated by RDS Analyst

In the multivariate analysis presented in Table 4, the facilitators remaining significantly associated with PrEP acceptability were: not having to pay for PrEP (*OR* = 2.59, 95%CI: 1.50–4.46), access to personal support and support around the use of PrEP (*OR* = 4.35, 95%CI: 3.26–5.80), drug accessibility at the level of MSM networks (*OR* = 9.82, 95%CI: 3.50–27.52). One barrier proved to be significant: the concern that “taking PrEP means that one puts himself at risk for HIV” (*OR* = 0.11, 95%CI: 0.04–0.30). Other associated factors were: age 20–34 years (*OR* = 24.50, 95%CI: 4.13–145.24), in comparison with < 20 years (*OR* = 6.23, 95%CI: 1.49–26.52); and divorced and / or widowed status (*OR* = 103, 95%CI: 22.69–469.86).

**Table 4:** Multivariate Analysis of PrEP acceptability according to MSM characteristics, facilitators and obstacles, Benin

	OR^**a**^	95%CI	***p***-value
**Age categories**			
< 20	1		
20–24	6.29	1.49–26.52	**0.01**
25–29	5.28	0,80-34,67	0.08
30–34	24.50	4.13–145.24	**0.0005**
≥ 35	0.58	0.11–2.99	0.51
**Marital Status**			
Married	1		
Single	2.03	0.63–6.52	0.23
Divorced or widowed	103.27	22.69–469.86	**< 0.0001**
De facto union	0.37	0.10–1.41	0.14
**Facilitators**			
**Not having to pay for PrEP**			
5	2.59	1.50–4.46	**0.0006**
(4 + 3 + 2 + 1)	1		
**Access to personal support and support around the use of PrEP**			
5	4.35	3.26–5.80	**< 0.0001**
(4 + 3 + 2 + 1)	1		
**Drug accessibility at the level of MSM networks**			
5	9.82	3.50–27.52	**< 0.0001**
(4 + 3 + 2 + 1)	1		
**Obstacles**			
**The concern that taking PrEP means that I put myself at risk for HIV**			
(5 + 4)	0.11	0.04–0.30	**< 0.0001**
(3 + 2 + 1)	1		

^a^ weighted odds ratios (probability weights generated by RDS Analyst)

## Discussion

Most of the MSM surveyed lived in Cotonou, the economic capital of Benin. In general, MSM were young adults, christians, single people with a good level of education, most of them exclusively homosexual. They mostly preferred daily PrEP (vs. PrEP on demand). They estimated that their HIV-related individual risk was not high. About half of them had knowledge about PrEP, but once well informed, most of them were willing to use it if made available in Benin. Young adults, as well as those divorced and / or widowed were more likely to use it. Factors that could ease this use were: not having to pay for PrEP, access to individual support and support around the use of PrEP, and drug availability within the MSM networks.

PrEP could lead to the reduction or even the abandonment of traditional HIV prevention methods with an increasing risk of HIV, as most of those who were willing to use PrEP also considered reducing their use of condoms, or even expanding the number of sexual partners.

These results have a lot of similarities with those observed in other contexts. On this same issue, other studies conducted in low- and middle-income countries [35–37] also described relatively young educated subjects, mostly homosexual who were willing to use PrEP. The level of PrEP knowledge and associated factors seems to vary by context. The level of PrEP knowledge among our participants was quite high and appeared to be similar to what was observed in Nigeria and Kenya [16, 17]. It is not always high in developing countries [38]. In one Indian cohort, none of the focus group participants had heard of the term “PrEP”, nor were they aware that antiretroviral treatment could be used to prevent HIV infection [39]. It was found that despite the low level of PrEP awareness, MSM in low-income countries are willing to use it if they are appropriately supported to address a range of individual, social, and structural barriers [38].

The acceptability level of PrEP that we found was quite high even though respondents were unfamiliar with this prevention strategy. It should be noted that at the beginning of each questionnaire administration, an accurate information note was read and explained to the participants. This measure made it possible for them not to have to decide on a strategy on which they did not have enough information. From this point of view, we can say that the level of acceptability obtained reflects the reality of MSM’s intention to use PrEP if made available.

PrEP acceptability level varies across countries and for various reasons: In Nigeria, 53.6% of MSM were aware of PrEP and 80.1% were willing to use PrEP in Nigeria [16]. In Kenya, 64.3% who had heard of PrEP but only 50% were willing to use it. And condom use with regular partners, improved self-efficacy in condom use, better perception of ability to use PrEP, history of STIs and membership of an LGBT organization were significantly associated with knowledge of PrEP [17].

In some studies on the intention to use PrEP, a high level of PrEP acceptability was sometimes related to the intention to enhance risky sexual behaviors, such as stopping condom use and increasing the number of unprotected sexual contacts [40–43]. No PrEP studies in non-MSM individuals, including heterosexual people of the general population and sex workers, have shown an increase in risky behaviors under PrEP [44–46].

Factors that could affect the acceptability of PrEP were also found by others [13, 15, 24]. Attributing a financial cost to PrEP will affect observance and therefore the expected efficiency [23]. Similarly, the medication accessibility at the MSM network level would be an asset in the adoption of PrEP. The use of the drug would be easier because the fear of stigmatization would be reduced. PrEP programs will need to rely on HIV health and care services designed for key populations so as to reduce stigma and facilitate entry and clients retention [47]. The concern that taking PrEP means that a MSM puts himself at risk for HIV was found to be an obstacle to PrEP use. Taking PrEP may encourage more risky behavior. This is described in several papers [48–51]. Sexual behavior has been assessed by some authors by collecting information on: the number of male sexual partners, sex outside the main relationship, frequency of unprotected insertive and receptive sex, condom use at last sex, anal sex with both primary and occasional partners [52]. It would be unfortunate to lose all the benefits made in adopting good prevention practices at the expense of PrEP use. Strategies to help PrEP users recognize the types of risk associated with the situation and adopt appropriate prevention practices, such as limiting the number of sexual partners and the availability of condoms, can be a valuable complement for advising on PrEP therapeutic adherence [53]. However, this issue needs further discussion because the use of PrEP has not always led to higher rates of HIV or STI acquisition [54]. Significant differences have not always been demonstrated in the incidence of STIs among MSM PrEP users, compared to non-users [55]. Regular monitoring of MSM users of PrEP will allow to treat their STIs quickly, which could even lead, in the long run, to a reduction in STIs [56]. PrEP prescribers have an important role to play in testing and treating STIs as the incidence may increase if PrEP is administered without these services [57].

Other factors associated with PrEP acceptability were age of the MSM and their marital status. In the present study, PrEP acceptability was higher in men aged 25–34 years compared the oldest and youngest age groups. Although only a small proportion of men was aged < 20, the use of this group as the reference category allowed to detect this interesting association. The relationship between age and the acceptability of PrEP is variously discussed in the literature. Some studies confirm this relationship [58–60]. Others describe acceptability as higher among young MSM reporting risky sexual behaviors [61–63]. Few studies have found a significant relationship between age and the acceptability of PrEP [64]. With respect to marital status, the strength of the association between divorced / widowed marital status and PrEP acceptability was quite high, mainly due to the small size in this group and to a lesser extent to the use of odds ratios instead of prevalence ratios.

Previous studies on MSM in Benin focused on describing their characteristics as well as HIV prevalence and incidence in that community [5, 65]. Our study is the first to address the issue of PrEP among MSM in Benin and one of the first in West Africa [66]. The recruitment technique used is recognized as adequate for collecting reliable data among hidden populations such as MSM. Potential biases that could be introduced by this technique were taken into account by fitting the data to probability weights generated according to the requirements of the RDS method [31].

Limits of this study lie in the possibility of social desirability bias and non-representativeness of the entire MSM community in Benin, even though the study was held in six large cities throughout the country. Social desirability biases may come from the fairly sensitive questions asked during face-to-face interviews with participants. Non-representativeness is mentioned because only cities with high concentration of MSM (and only those who come out), have been selected for this study. However, in practice, in the current context of Benin, only MSM known as such are accessible and they represent only a small proportion of all MSM. This study does not consider the most hidden MSM, whose proportion is likely to be high in a context of important stigmatization of MSM, as in many African countries. Beyond these limits, the results obtained could be very useful in the implementation process of PrEP strategy among MSM in Benin.

## Conclusion

Not all MSM knew about PrEP in Benin, but, after being informed about it, almost all participants were willing to use PrEP if made available. The free availability of the drug and its accessibility in the MSM networks could facilitate the use of PrEP among MSM in Benin. The implementation of the PrEP strategy will have to consider these factors to ensure its success in Benin. Although some MSM may reduce condom use and increase the number of sexual partners and intercourses if using PrEP, this should not hamper its implementation. When available, prescribers should provide informed support to MSM in choosing their HIV prevention options (condom and / or PrEP).

## Data Availability

The datasets used and/or analysed during the current study are available from the corresponding author on reasonable request.

## Abbreviations

HIV: Human Immunodeficiency Viruses
MSM: Men who have Sex with Men
PrEP: Pre-exposure Prophylaxis
RDS: Respondent Driven Sampling
WHO: World Health Organization;
STI: Sexually Transmitted Infections
LGBT: Lesbian, Gay, Bisexual, Transgender
